# Risk factors for interpersonal violence: an umbrella review of meta-analyses

**DOI:** 10.1192/bjp.2018.145

**Published:** 2018-10

**Authors:** Seena Fazel, E. Naomi Smith, Zheng Chang, John Richard Geddes

**Affiliations:** 1 Professor of Forensic Psychiatry, Department of Psychiatry, University of Oxford, UK; 2 Registrar in General and Older Adult Psychiatry, Department of Psychiatry, University of Oxford, UK; 3 Assistant Professor, Department of Medical Epidemiology and Biostatistics, Karolinska Institutet, Sweden; 4 Professor of Epidemiological Psychiatry, Department of Psychiatry, University of Oxford and Oxford Health NHS Foundation Trust, UK

**Keywords:** Violence, risk factor, meta-analysis, aggression

## Abstract

**Background:**

Interpersonal violence is a leading cause of morbidity and mortality. The strength and population effect of modifiable risk factors for interpersonal violence, and the quality of the research evidence is not known.

**Aims:**

We aimed to examine the strength and population effect of modifiable risk factors for interpersonal violence, and the quality and reproducibility of the research evidence.

**Method:**

We conducted an umbrella review of systematic reviews and meta-analyses of risk factors for interpersonal violence. A systematic search was conducted to identify systematic reviews and meta-analyses in general population samples. Effect sizes were extracted, converted into odds ratios and synthesised, and population attributable risk fractions (PAF) were calculated. Quality analyses were performed, including of small study effects, adjustment for confounders and heterogeneity. Secondary analyses for aggression, intimate partner violence and homicide were conducted, and systematic reviews (without meta-analyses) were summarised.

**Results:**

We identified 22 meta-analyses reporting on risk factors for interpersonal violence. Neuropsychiatric disorders were among the strongest in relative and absolute terms. The neuropsychiatric risk factor that had the largest effect at a population level were substance use disorders, with a PAF of 14.8% (95% CI 9.0–21.6%), and the most important historical factor was witnessing or being a victim of violence in childhood (PAF = 12.2%, 95% CI 6.5–17.4%). There was evidence of small study effects and large heterogeneity.

**Conclusions:**

National strategies for the prevention of interpersonal violence may need to review policies concerning the identification and treatment of modifiable risk factors.

**Declarations of interest:**

J.R.G. is an NIHR Senior Investigator. The views expressed within this article are those of the authors and not necessarily those of the NHS, the NIHR or the Department of Health and Social Care.

Interpersonal violence is among the most important preventable causes of premature mortality and morbidity. Excluding war, it leads to around 410 000 deaths per year and is the 19th most common cause of death globally.^1^ Morbidity is also substantial, although there are large variations, it is in the top five causes of disability-adjusted living years in central and tropical Latin America, and southern Sub-Saharan Africa.^2^ Trends in violence vary depending on the outcome used: decreases in violence-related mortality have been reported from 2000 to 2015,^1^ whereas morbidity has remained unchanged.^1^^,^^2^

Public health has moved toward a prevention model for violence,^3^ and influential World Health Organization reports have focused on delineating risk factors.^4^ Identifying modifiable risk factors could potentially reduce risks and assist in developing interventions. However, these reports are limited by being narrative reviews of the evidence without quantitative methods to evaluate the strength, quality and consistency of risk factors.

To address limitations in previous work and provide an overview, we conducted an umbrella review of the evidence from existing systematic reviews and meta-analyses on risk factors for violence.

## Methods

No specific ethical approval was required for this research as it was a synthesis of secondary data from published sources.

### Search strategy

The systematic search strategy was prospectively registered on PROSPERO^5^ (registration number CRD42014010400). The original search incorporated both risk factors for violence and suicide, and this paper reports the violence search. Three databases were searched from their start dates until January 2018: PsycINFO (1 January 1806 to 5 January 2018), Medline (1 January 1946 to 5 January 2018) and Global Health (1 January 1973 to 5 January 2018), supplemented by targeted searches on Google Scholar (1 January 2004 to 5 January 2018) and PubMed (1 January 1996 to 5 January 2018).

Keywords for violence (violen*, crim*, offen*, antisocial and delinq*) were combined with search terms for risk factors (risk, predict*’) and publications (meta*, systematic review). Citations and reference lists of relevant reviews were hand-searched. Targeted searches were used to identify additional studies by first author names and specific risk factors that were not identified in our initial search (including developmental disorders).

### Study eligibility

Eligible studies were meta-analyses or systematic reviews that examined risk factors for violence in the general population, and provided effect sizes and data to calculate 95% confidence intervals. We aimed to measure interpersonal violence and included a broad range of violence outcomes, such as assault, violent crime and sexual violence. Although this is a broad scope, we aimed to include only those reviews that used some measure of interpersonal violence as outcome (so that verbal aggression, minor criminality and antisocial behaviour were excluded). Published and unpublished reviews in any language were considered.

Excluded studies were those with methodologies other than a meta-analysis or systematic review, such as individual case–control or cohort studies. As the primary research question was risk factors in the general population, reviews that investigated selected populations, such as prisoners or those with a specific diagnosis, were excluded. Reviews that focused on reoffending risks or those examined interventions for violence were also excluded.^6^^–^^8^ If more than one eligible review was found on the same risk factor, the most recent one was included.

### Data extraction

Data were extracted with a standardised form. Reported effect sizes with 95% confidence intervals were recorded with other key information. Separate effect sizes for gender, the effect size of the largest study included in each meta-analysis and the effect size for the different study designs were extracted. When these data were not recorded, we corresponded directly with authors. Extracted data were independently cross-checked by a post-doctoral researcher (Z.C.), and any queries were resolved by discussion with the project supervisor (S.F.).

### Statistical analyses

As the reporting of effect sizes varied between studies (including odds ratios, Cohen's *d*, correlation coefficients, relative risks and standardised mortality ratios), they were converted to comparable measures. For the primary outcome, all effect sizes were converted to odds ratios (for selected formulae, see Supplementary Appendix 1 available at https://doi.org/10.1192/bjp.2018.145). For those reported as Cohen's *d*, log-transformed odds ratios were calculated.^9^^–^^11^ Effect sizes reported as correlation coefficients were converted first to Cohen's *d* and then to log-transformed odds ratios. Odds ratios were categorised as follows: weak, 1.0–1.5; moderate, 1.6–2.5; strong, 2.6–9.9 and very strong, ≥10.0.^12^

### Categorisation of risk factors and outcome measures

Risk factors and outcome measures were qualitatively analysed after the search, and common categories were identified. We identified distinct categories of outcome measures (any interpersonal violence, intimate partner violence, sexual violence and homicide) that were reported separately. Meta-analyses with other related outcome measures, such as aggression and hostility, were reported as secondary outcomes in Supplementary Appendix 2.

### Population attributable risk fractions

Population attributable risk fractions (PAFs) indicate the proportion of an outcome that would theoretically not occur in a population if a given risk factor was eliminated, assuming causality between risk factor and outcome. We estimated the proportion of cases that could be attributed to each risk factor in the general population (see Supplementary Appendix 1 for formulae). Although causal inferences were not possible for some risk factors, PAFs provide a measure of the maximum possible effect that each risk factor has at a population level by taking into account the risk factor's prevalence.^13^ Thus, if a risk factor has a large effect size but low prevalence, its effect at a population level will be lower than a risk factor with low or moderate effect but a high prevalence.

### Tests of quality of evidence

Reviews were assessed for quality by various approaches. First, we scored the Assessing the Methodological Quality of Systematic Reviews (AMSTAR) tool.^14^ Scores of 0–3 are considered low, 4–7 are medium and 8–11 are high.^12^ Second, we compared the effect size for the largest included study in each meta-analysis with the overall quoted meta-analysis effect size. Results where the largest included study effect size (assumed to be the most accurate) was close to the overall meta-analysis effect size were deemed to be more precise.^15^ Third, we calculated ratios between overall meta-analysis effect size and that of the largest included study in each meta-analysis. A meta-analysis overall effect size/largest included study effect size ratio of more than one indicates a larger effect size in the meta-analyses compared with its largest included study, and is an indication of bias.^16^ Fourth, a comparison was made between meta-analyses' overall effect size and the number of cases included in each meta-analysis (meta-analyses with large sample sizes were deemed to be more precise^15^), when sufficient data were available. Fifth, we assessed the relationship between study design and effect size. Where sufficient data were available, results were extracted for pooled overall effect sizes of prospective studies alone and compared with overall meta-analysis' effect sizes. Finally, we presented prediction interval calculations for risk factors. Prediction intervals provide an estimate of the ranges in which future observations will fall. Risk factors with prediction intervals that did not cross the null value were deemed to be of higher quality. Those that cross the null value suggest that they may not be significant if tested in a new population.^17^ To summarise these quality tests, a scoring system was developed, which also included between-study heterogeneity (with *I*^2^ <50% categorised as low heterogeneity) and whether adequate adjustments for confounders was conducted (see Table 1 for details on the scoring system). All analyses were performed with STATA-IC version 13.

**Table 1:** Top five risk factors for interpersonal violence ranked by quality of evidence

Risk factor	Prediction interval excludes null value	*P*-value	Heterogeneity	Number of cases > 1000	Small study effects	Confounders adjusted	Total score (maximum core = 6)
Antisocial personality disorder	Yes	0.01	Low (*I*^2^ < 50%)	Yes	No	Yes	5
Bipolar disorder	Yes	<0.001	High (*I*^2^ > 50%)	Yes	Yes	Yes	4
Schizophrenia	Yes	<0.001	High (*I*^2^ > 50%)	Yes	Yes	Yes	4
Non-schizophrenia psychoses	Yes	<0.001	High (*I*^2^ > 50%)	Yes	Yes	Yes	4
Victimisation by bullying	No	0.042	Low (*I*^2^ < 50%)	Not reported	No	Yes	4

Scores: Prediction interval excluding null value = 1; *P*-value <0.05 for random effects model = 1; low heterogeneity (*I*^2^ < 50%) = 1; number of cases > 1000 = 1; no evidence to suggest small study effects = 1; confounders adjusted for = 1.

## Results

**Fig. 1:**
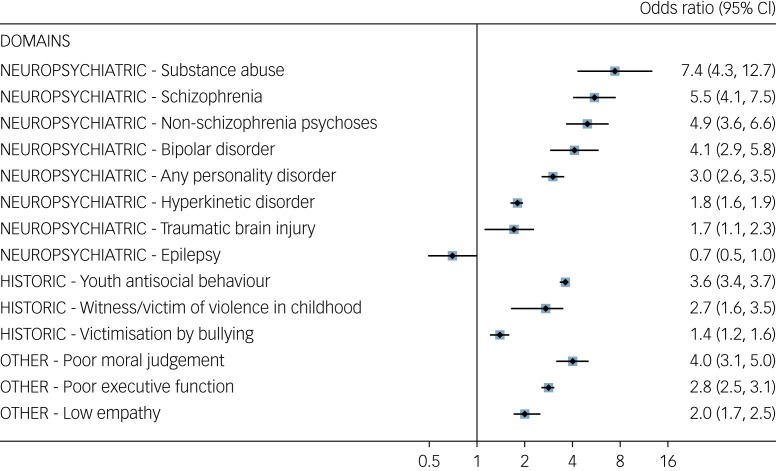
Effect sizes of risk factors (identified in meta-analyses) for interpersonal violence, ranked by strength of association and subcategory. Adjusted odds ratios were used when possible.

Twenty-two meta-analyses on risk factors for violence (Supplementary Appendix 3) were identified.^18^^–^^39^ This included information from over 120 000 individuals from 1139 individual studies across 14 different countries. Risk factors were grouped into broad categories or domains of neuropsychiatric, historic and other. Because of high heterogeneity and non-comparability, results were not further pooled. The largest effect sizes for violence were found in the neuropsychiatric category (Fig. 1), with substance misuse ranking most highly. Antisocial personality disorder had the strongest link to violence within the category of personality disorders.

Some childhood and adolescent factors were important (particularly youth antisocial behaviour). Four meta-analyses examined parental factors that were associated with violence^20^^,^^26^^,^^31^^,^^35^ (Supplementary Appendix 4). These factors included poor attachment to parents, parental incarceration, antisocial attitudes in parents and more general problems within the family.

### Intimate partner violence

Six meta-analyses focused on intimate partner violence.^32^^–^^37^ Two risk factors overlapped with risk factors for any interpersonal violence, namely substance misuse and exposure to violence. Other risk factors for intimate partner violence appeared to be specific to relationships, such as marital dissatisfaction and previous abuse by one partner toward the other (Supplementary Appendix 5).

### Sexual violence and homicide

Two reviews provided data for risk factors for sexual violence alone,^38^^,^^39^ and only one review provided separate risk estimates for homicide^21^ (Supplementary Appendix 6). Risk factors for sexual violence broadly overlapped with risk factors for any interpersonal violence. Data were more limited for the homicide review although two neuropsychiatric risk factors (schizophrenia and substance misuse) overlapped with interpersonal violence.

### Risk factors stratified by gender

Where possible, results were stratified by gender (Supplementary Appendix 7). Effect sizes for women appeared to be larger than for men for all neuropsychiatric violence risk factors.

### PAFs

**Fig. 2:**
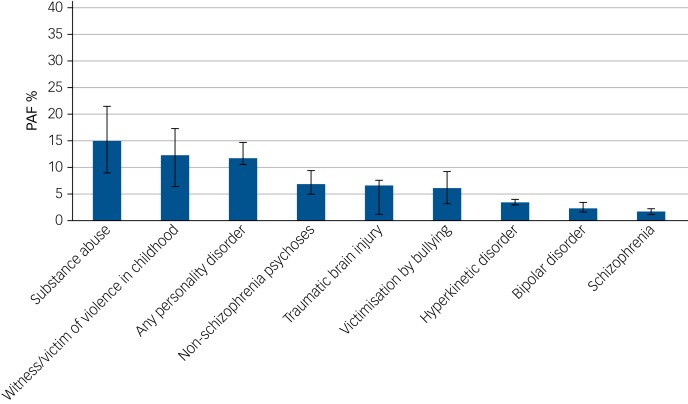
Population attributable fractions (PAFs) of risk factors (identified in meta-analyses) for interpersonal violence.

Although PAFs assume causality, they provide an estimate of the maximum possible effect that removing a risk factor could have, and PAFs for individual risk factors may overlap and add up to more than 100%.^40^ The highest PAFs for violence were substance misuse, witnessing or being a victim of violence in childhood, and personality disorder (Fig. 2).

### Other reviews

We identified a further 13 systematic reviews and meta-analyses that provided additional information. For violence, these were for the secondary outcomes of aggression and hostility rather than interpersonal violence (Supplementary Appendix 2). Risk factors for aggression included two main themes: biological factors (serotonin and testosterone levels, heart rate, genetic influences and electrodermal activity) and witnessing violence (e.g. being exposed to television violence and violent videogames). Negative findings included the lack of evidence for candidate genes associated with aggression in a meta-analysis and field synopsis of 185 studies of the field.^41^

### Quality assessments

**Fig. 3:**
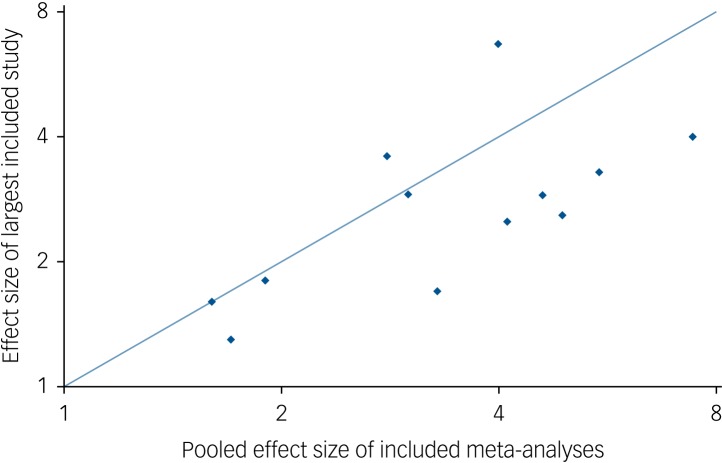
A comparison of pooled effect size of included meta-analyses and the effect size of the largest included study in these individual meta-analyses.

Despite mostly high scores on AMSTAR, other analyses found indications of poorer quality. There were small study effects and around 60% of reviews had overall effect sizes larger than the effect size quoted in each meta-analysis' largest included study (Fig. 3; ratios in Supplementary Appendix 8). There was no statistically significant correlation between meta-analyses' overall effect size and the number of cases included in each meta-analysis, when sufficient data were available. Of the 12 included risk factors, seven were found to exclude the null value using prediction intervals (Supplementary Appendix 9).

Three meta-analyses enabled investigation of study design.^21^^,^^24^^,^^25^ One review, which examined being bullied as a risk factor, reported a lower pooled effect size for prospective studies (odds ratio 1.8, 95% CI 1.3–2.3 versus overall odds ratio 4.9, 95% CI 2.1–11.2).^24^ Two other reviews did not find statistically significant differences by study design (one of which examined prospective studies versus case–control designs in schizophrenia,^21^ and the other examined nested case–control versus others in childhood witnessing of violence^25^).

Overall, using a scoring system (with a maximum of six) based on quality indicators and a threshold of four or above for high-quality studies, seven risk factors for violence met these criteria. None of the risk factors for intimate partner violence or sexual offending met this quality threshold (see Table 1 for top five risk factors based on quality scores; see Supplementary Appendix 10 for a full list and explanation of the scoring system).

## Discussion

We have presented an overview of risk factors for interpersonal violence from 22 meta-analyses based on over 120 000 individuals. We have presented associations, PAFs and measures of evidence quality, and investigated risk factors for related outcomes of homicide, intimate partner violence and sexual offending. To our knowledge, this is the first quantitative meta-review of the field. In addition, novel features include bringing together relative risks and estimates of population effect, using tests of methodological quality to determine the strength of the underlying evidence, and the breadth of the outcomes and the ability to compare effect sizes between them.

There were three principal findings. First, based on relative risk, the strongest risk factors were typically in the neuropsychiatric domain. Second, in terms of population effect, there was some overlap with factors that had the strongest relative effects, with substance use disorders, schizophrenia and personality disorders having high PAFs and relative risks. Third, the overall quality of the underlying evidence was not strong, with the majority of reviews demonstrating small study effects and large heterogeneity. By focusing on risk factors, this umbrella review has identified individual-level determinants. Socioeconomic causes of violence will rely on ecological studies that were not included.

A number of implications arise from this work. First, it suggests that many important risk factors for violence are modifiable, and public health can realistically include substantial reductions globally if these factors are confirmed in treatment trials as causal.^42^ Second, violence prevention strategies should incorporate guidelines and targets for the identification, assessment and treatment of psychiatric disorders. However, diagnostic categories themselves are not sole treatment goals, and active symptoms and comorbidities, which mediate the above-reported associations with violence, should also be targeted. Our findings challenge the current view of criminology as a field that appears to under-recognise mental health in the aetiology of violent crime.^43^ In contrast, this umbrella review found no relevant meta-analyses that were among the top five risk factors in terms of quality for socioeconomic variables, and only one for a psychosocial factor (moral judgement). One possible explanation is that the focus of many included reviews were neuropsychiatric conditions rather than socioeconomic factors. In addition, within the former, the variation in socioeconomic factors is limited, and thus studying their effects will require more general population samples.

At the same time, it should be noted that criminal history variables are among the strongest for individuals with psychiatric disorders, underscoring the need to strengthen the relationship between criminal justice and mental health services to manage future risks. Third, on a population level, antisocial personality disorder is an important risk factor for violence, and more research on links between such disorders and these outcomes is warranted. Although little evidence exists to suggest that the underlying personality disorders are treatable, some common symptoms arising from them are modifiable.^44^ Another risk factor identified, which has been less widely discussed, is witnessing or being a victim of violence in childhood. The mechanism for how this contributes to adult violence perpetration needs examination, and may provide targets for intervention. Nevertheless, it suggests that interventions in childhood and adolescence for antisocial behaviour should consider any such history and broaden treatments for victims to include children who have witnessed violence. Finally, research should focus on longitudinal studies, investigate sources of heterogeneity and improve adjustment for confounding. Sibling controls are one powerful approach to do so,^45^ and can provide important evidence as they account for familial confounding (early environmental and genetic factors). Ultimately, strong evidence of causal inference for identified risk factors will need to be tested in trials. However, many trials in this area may not be feasible for practical and ethical reasons, and quasi-experimental designs (such as observational studies using family designs and natural experiments) will play an important role in developing the evidence base.

Limitations of the current meta-review include the possibility that the included meta-analyses have been superseded by more recent, high-quality individual studies. For example, the reviews on traumatic brain injury and schizophrenia are from 2009.^21^^,^^22^ However, both of these have been confirmed by more recent, large population-based studies.^46^ In relation to traumatic brain injury, a large Swedish population and sibling comparison investigation found robust links with violent crime after adjustment for sociodemographic confounders,^47^ and an Australian study also found a link when violent crime (as opposed to any crime) was used as an outcome (with additional adjustment for previous criminality).^48^ In addition, how violence was operationalised was necessarily heterogeneous, reflecting the lack of a consensus in the field for the best outcome.^49^ Importantly, although these will alter prevalence of outcomes, they does not appear to affect risk estimates as the prevalence of outcomes is consistently reported in the cases (subgroups defined by exposure to a particular risk factor) and general population controls.

How might treatment reduce violence? One approach is simply to target and treat underlying psychiatric disorders as well as symptoms and other mediators of risk. Randomised controlled trials provide little evidence for this approach as they are not usually powered or designed to investigate rare outcomes. Observational data provide stronger support for antipsychotic medication reducing violence risk,^50^ and are important sources of evidence when randomised controlled trials are not feasible. For example, clozapine may have specific violence-reducing effects^51^ and psychological therapies that specifically target aggression could also be considered. There is some evidence for structured group therapy in drug-using offenders to prevent reoffending.^52^ Screening for violence risk in selected populations^53^ needs further research to clarify its potential role, including use of trial methodology. Targeting high-risk groups, such as released prisoners and individuals with antisocial personality disorder, should be prioritised for future intervention research. Treatments in childhood and adolescence require improvement.^54^ In addition, preventative approaches should be developed to address the potential importance of the two childhood risk factors that we have identified: being bullied and witnessing or experiencing violence.

## Supplementary material

For supplementary material accompanying this paper visit https://doi.org/10.1192/bjp.2018.145. click here to view supplementary material
