# Supplementary material for: Risk factors for interpersonal violence: an umbrella review of meta-analyses
Source: Br J Psychiatry. 2018 Oct;213(4):609–14. doi: 10.1192/bjp.2018.145 (PMC6157722; doi:10.1192/bjp.2018.145)
Supplement: Supplementary file 1 [file S0007125018001459sup001.docx]

**Supplementary Material** **- BJPsych-18-0043 - Appendices**

# *Appendix 1 – Formulae used*

1. Converting Cohen’s d to Odds Ratio:

**Cohen’s d = Log OddsRatio(√3/∏)**

**Or, OR = e^∏d/√3^**

1. Converting correlation coefficient (‘r’) to Odds Ratio:

First r was converted to Cohen’s d as follows:

**Cohen’s d = 2r / √(1-r^2^)**

The Cohen’s d value was then used to calculate Odds Ratio from formula:

**Cohen’s d = LogOddsRatio(√3/∏)**

**Or, OR = e^∏d/√3^**

1. Converting Odds Ratio to Relative Risk:

**RR = OR/(1-P_0_+(P_0_ x OR)**

*Where P_0_ = baseline risk or prevalence*

1. Population attributable fraction:

**PAF = [P_0_ (RR-1)]/[(1+ P_0_ (RR-1)]**

*Where P_0_ is the proportion of exposed subjects in the study population*

The above are based on reference numbers: 9, 10 and 11 in main manuscript

# *Appendix 2 - Risk Factors with Outcome Measures Related to Interpersonal Violence*

| Risk Factor | Outcome Measure | Review Type | Summary |
| --- | --- | --- | --- |
| Post-traumatic stress disorder ^i^ | Anger and hostility | Meta-analysis | Weighted mean effect size r = 0.5 |
| Exposure to violence in the form of video games, television and film ^ii^ | Aggression | Meta-analysis | Overall effect size was, r = 0.1 |
| Exposure to media violence ^iii^ | Aggression and criminal aggression | Meta-analysis | Authors unable to calculate effect size for criminal aggression. Effect size for aggression, r = 0.2 |
| Exposure to violent video-games ^iv^ | Aggression | Meta-analysis | “Causal risk factor” – Overall r = 0.2 |
| Genetic influences ^v^ | Antisocial behaviour | Meta-analysis | r = 0.3 - Variance due to additive genetic influences, |
| Individual gene studies ^vi^ | Violence and aggression | Meta-analysis | No candidate gene studies were associated with violence. |
| Central serotonin function ^vii & viii^ | Aggression and antisocial behaviour | Meta-analyses (2) | r = - 0.1 for aggression  d = - 0.5 for antisocial behaviour |
| Low resting heart rate ^ix, x,xi^ | Antisocial behaviour and aggression | Meta-analyses (3) | d = -0.2 (SE 0.39, p < .0010.3-0.5) for low resting heart rate.  d = 0.4 (0.3-0.5) for low resting heart rate.  d = 0.1 (0.1-0.3) for resting electrodermal activity  d = 0.1 (-0.0-0.2) for heart rate during stressor – Authors conclude heart rate reactivity is not significantly associated with aggression.  d = - 0.4 (-.5- -0.4) for resting heart rate and levels of antisocial behaviour in children and adolescents  d = - 0.8 (-0.9 - -0.6) for heart rate during stressor in children and adolescents. |
| Impairments in P300 event-related potential and P300 latencies ^xii^ | “Antisocial” or “psychopathic” behaviour | Meta-analysis | d = 0.3 (0.2-0.3) - reduced P3 amplitudes  d = 0.1 (0.0-0.3) - longer P3 latencies |
| Increased testosterone levels ^xiii^ | Aggressive behaviour | Meta-analysis | r = 0.1 (-0.3 – 0.7) |

*References*

1. Orth, U. and E. Wieland. Anger, hostility, and posttraumatic stress disorder in trauma-exposed adults: A meta-analysis. *Journal of Consulting and Clinical Psychology* **74(4**), 698 (2006).
2. Savage, J., & Yancey, C. The effects of media violence exposure on criminal aggression a meta-analysis. *Criminal Justice and Behavio*r **35(6)**, 772-791 (2008).

Ferguson, C. J., & Kilburn, J. The public health risks of media violence: A meta-analytic review. *The Journal of pediatrics* **154(5)**, 759-763 (2009).

Anderson, C. A. et al. Violent video game effects on aggression, empathy, and prosocial behavior in eastern and western countries: a meta-analytic review. *Psychological bulletin* **136(2)**, 151 (2010).

Rhee, S. H., & Waldman, I. D. Genetic and environmental influences on antisocial behavior: a meta-analysis of twin and adoption studies. *Psychological bulletin*, **128(3)**, 490 (2002).

Vassos, E., et al. Systematic meta-analyses and field synopsis of genetic association studies of violence and aggression. *Molecular psychiatry* **19(4)**, 471-477 (2013).

1. Moore, T. M., Scarpa, A., and Raine, A. A meta‐analysis of serotonin metabolite 5‐HIAA and antisocial behavior. *Aggressive behavior* **28(4)**, 299-316 (2002).

Duke, A. A., et al. Revisiting the serotonin–aggression relation in humans: A meta-analysis. *Psychological bulletin* **139(5)**, 1148-72 (2013).

Lorber, M. F. Psychophysiology of aggression, psychopathy, and conduct problems: a meta-analysis. *Psychological bulletin* **130(4)**, 531 (2004).

Ortiz, J. and A. Raine. Heart rate level and antisocial behavior in children and adolescents: A meta-analysis. *Journal of the American Academy of Child & Adolescent Psychiatry* **43(2)**, 154-162 (2004).

[Portnoy](https://scholar.google.co.uk/citations?user=f5LFVwQAAAAJ&hl=en&oi=sra) J., Farrington D.P., [Resting heart rate and antisocial behavior: An updated systematic review and meta-analysis](https://www.sciencedirect.com/science/article/pii/S135917891500035X). *Aggression and violent behavior*, 2015 – Elsevier.

Gao, Y. and Raine, A. P3 event-related potential impairments in antisocial and psychopathic individuals: A meta-analysis. *Biological psychology* **82(3)**, 199-210 (2009).

1. Book, A. S., Starzyk, K. B. and Quinsey, V. L. The relationship between testosterone and aggression: A meta-analysis. *Aggression and Violent Behavior* **6(6)**, 579-599 (2001).

# *Appendix 3 – PRISMA flow diagram of systematic search strategy for risk factors for violence*

# *Appendix 4 - Effect sizes of parental risk factors for violence*


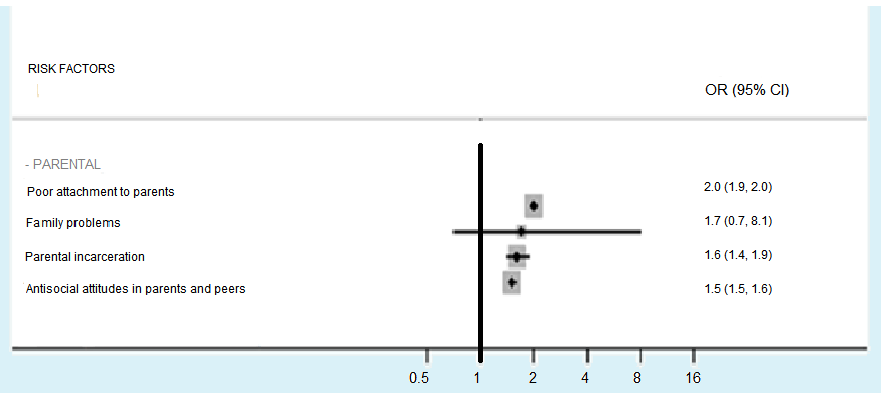


**Figure – Effect sizes of parental risk factors for violence ~~Forest~~**

Note: OR=odds ratio, CI=confidence interval. Adjusted ORs were used when possible.

# *Appendix 5 - Effect sizes of risk factors for intimate partner violence*

**
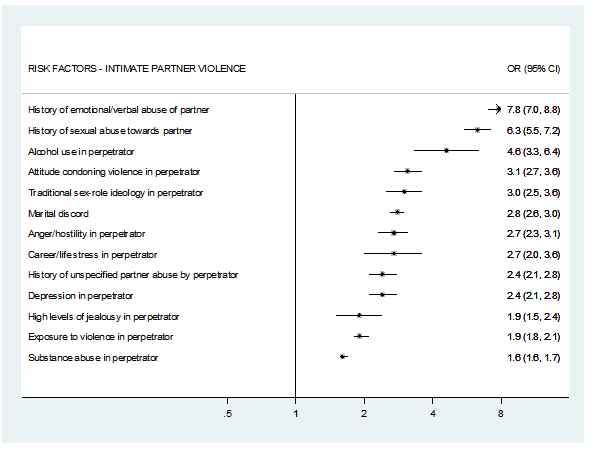
Figure – Effect sizes of risk factors for intimate partner violence ~~Forest~~**

Note: OR=odds ratio, CI=confidence interval. Adjusted ORs were used when possible.

# *Appendix 6 - Effect sizes of risk factors for sexual violence and homicide*


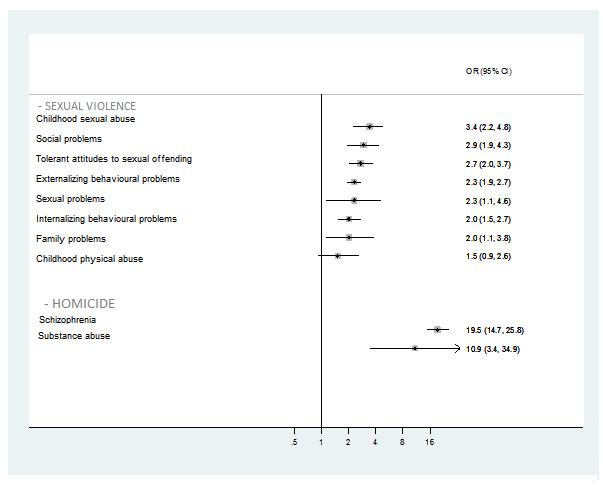


**Figure – Effect sizes of risk factors for sexual violence and homicide**

Note: OR=odds ratio, CI=confidence interval. Adjusted ORs were used when possible.

*Appendix 7 - Meta-review of risk factors for violence stratified by gender*

**
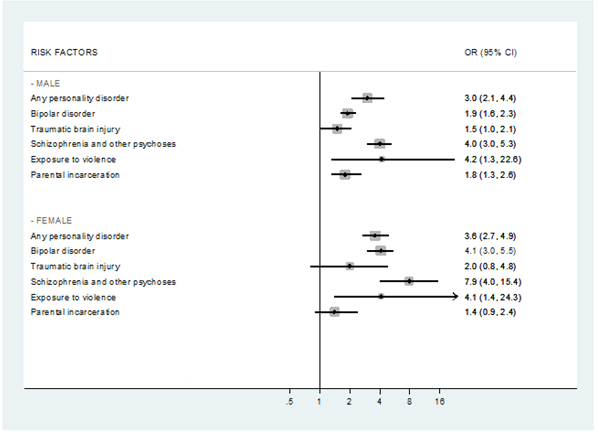
Figure – Meta-review of risk factors for violence stratified by gender**

Any personality disorder

Bipolar disorder

Traumatic brain injury

Schizophrenia and other psychoses

Exposure to violence

Parental incarceration

Any personality disorder

Bipolar disorder

Traumatic brain injury

Schizophrenia and other psychoses

Exposure to violence

Parental incarceration

Note: OR=odds ratio, CI=confidence interval. Adjusted ORs were used when possible.

# *Appendix 8 – Comparison of meta-analyses’ effect sizes*

Note: ES=effect size

| **Study** | **Meta-analysis ES (O)*** | **Largest Study ES (E)**** | **Observed/expected Ratio** |
| --- | --- | --- | --- |
| **Yu, 2012** | 3.0 | 2.9 | 1.0 |
| **Fazel, 2010** | 4.1 | 2.5 | 1.6 |
| **Fazel, 2009** | 1.7 | 1.3 | 1.3 |
| **Fazel, 2009** | 0.7 | 0.7 | 1.0 |
| **Fazel, 2009** | 7.4 | 4.0 | 1.9 |
| **Fazel, 2009** | 5.5 | 3.4 | 1.6 |
| **Fazel, 2009** | 4.9 | 2.6 | 1.9 |
| **Ttofi, 2012** | 1.4 | 1.3 | 1.2 |
| **Wilson, 2009** | 2.7 | 1.2 | 2.3 |
| **Murray, 2012** | 1.6 | 1.6 | 1.0 |
| **Stamms, 2006** | 4.0 | 6.7 | 0.6 |
| **Morgan, 2000** | 2.8 | 3.6 | 0.8 |
| **G-Gonzalez, 2006** | 4.6 | 2.9 | 1.6 |

**Table – A comparison of meta-analyses’ overall effect size (‘O’ or ‘observed’) versus effect size of meta-analyses’ largest included study effect size (‘E’ or ‘expected’).**

# *Appendix 9 – Effect sizes for risk factors with prediction intervals*


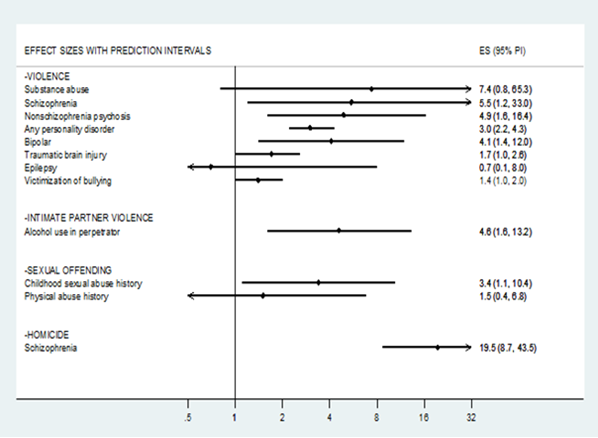


**Figure – Effect sizes for risk factors with prediction intervals**

Note: prediction intervals that exclude the null value are deemed to be of higher quality

# *Appendix 10 – Full Details of Quality Analysis Tables*

Scores: Prediction interval excluding null value = 1; p-value less than 0.05 for random effects model = 1; low heterogeneity (I^2^ <50%) = 1; case number > 1000 = 1; no evidence to suggest small study effects = 1; confounders adjusted for = 1.

| Risk Factor | Outcome Category | Prediction Interval Excludes Null Value | p-Value | Heterogeneity | Number of Cases > 1000 | Small Study Effects | Confounder Adjusted | Total Score (maximum score = 6) |
| --- | --- | --- | --- | --- | --- | --- | --- | --- |
|  |  |  |  |  |  |  |  |  |
| Substance abuse | Violence | No | 0.001 | High | Yes | Yes | Yes | 3 |
| Schizophrenia | Violence | Yes | < 0.001 | High | Yes | Yes | Yes | 4 |
| Nonschizophrenia psychoses | Violence | Yes | < 0.001 | High | Yes | Yes | Yes | 4 |
| Any personality disorder | Violence | Yes | 0.309 | Low | Yes | No | Yes | 5 |
| Bipolar disorder | Violence | Yes | < 0.001 | High | Yes | Yes | Yes | 4 |
| Traumatic brain injury | Violence | No | 0.585 | Low | Yes | Yes | Yes | 3 |
| Hyperkinetic disorder | Violence | No | < 0.01 |  | Yes |  | Yes | 3 |
| Epilepsy | Violence | No | 0.779 | Low | No | No | Yes | 3 |
| Youth antisocial behaviour | Violence |  |  |  | Yes |  | Yes | 2 |
| Victimization of bullying | Violence | No | 0.042 | Low |  |  | Yes | 4 |
| Exposure to violence | Violence |  |  | High | Yes |  | Yes | 2 |
| Poor attachment to parents | Violence |  |  | High | Yes |  | Yes | 2 |
| Parental incarceration | Violence |  | < 0.01 | High | Yes |  | Yes | 3 |
| Antisocial attitudes in parents and peers | Violence |  | < 0.01 | High | Yes |  | Yes | 3 |
| Family problems | Violence |  |  | High | Yes |  | Yes | 2 |
| Poor executive function | Violence |  |  | High | Yes | No | Yes | 4 |
| Poor moral judgement | Violence |  | < 0.001 | High | Yes | No | Yes | 4 |
| Low empathy | Violence |  |  | High | Yes |  | Yes | 2 |

| Risk Factor | Outcome Category | Prediction Interval Excludes Null Value? | p-Value | Low or High Heterogeneity | Number of Cases > 1000 | Evidence to Suggest Small Study Effects? | Confounder Adjusted for? | Total Score (maximum score = 6) |
| --- | --- | --- | --- | --- | --- | --- | --- | --- |
| Alcohol abuse | IPV | Yes | < 0.001 | High |  |  | Yes | 3 |
| History of emotional/verbal abuse of partner | IPV |  |  |  |  |  | Yes | 1 |
| History of sexual abuse towards partner | IPV |  |  |  |  |  | Yes | 1 |
| Attitude condoning violence in perpetrator | IPV |  |  |  |  |  | Yes | 1 |
| Traditional sex-role ideology in perpetrator | IPV |  |  |  |  |  | Yes | 1 |
| Marital discord | IPV |  |  |  |  |  | Yes | 1 |
| Anger/hostility in perpetrator | IPV |  |  |  |  |  | Yes | 1 |
| Career/life stress in perpetrator | IPV |  |  |  |  |  | Yes | 1 |
| History of unspecified partner abuse by perpetrator | IPV |  |  |  |  |  | Yes | 1 |
| Depression in perpetrator | IPV |  |  |  |  |  | Yes | 1 |
| High levels of jealousy in perpetrator | IPV |  |  |  |  |  | Yes | 1 |
| Exposure to violence in perpetrator | IPV |  |  |  |  |  | Yes | 1 |
| Substance abuse in perpetrator | IPV |  |  |  |  |  | Yes | 1 |
| Childhood sexual abuse | Sexual offending | Yes |  | High |  |  | Yes | 2 |
| Social problems | Sexual offending |  |  | High |  |  | Yes | 1 |
| Tolerant attitudes to sexual offending | Sexual offending |  |  |  |  |  | Yes | 1 |
| Externalizing behavioural problems | Sexual offending |  |  |  |  |  | Yes | 1 |
| Sexual problems | Sexual offending |  |  |  |  |  | Yes | 1 |
| Internalizing behavioural problems | Sexual offending |  |  |  |  |  | Yes | 1 |
| Family problems | Sexual offending |  |  |  |  |  | Yes | 1 |
| Physical abuse in childhood | Sexual offending | No |  | High |  |  | Yes | 1 |
| Schizophrenia | Homicide | Yes | \|  \| \| --- \|   0.042 | High |  | No | Yes | 2 |
| Substance abuse | Homicide |  | 0.001 | High |  | No | Yes | 1 |
